# Lea’s Series of Pocket Text-Books: Hayden on Venereal Diseases

**Published:** 1902-07

**Authors:** 


### Books and Magazines.

LEA'S SERIES OF POCKET TEXT-BOOKS: HAYDEN ON VENEREAL DISEASES. A Pocket Text-book of Venereal Diseases. For students and practitioners. By James R. Hayden, M. D., Chief of Clinic and Instructor in Venereal and Genito-Urinary Diseases in the College of Physicians and Surgeons, New York, Etc. New (3rd) edition, thoroughly revised. In one handsome 12mo. volume of 304 pages with 66 engravings. Cloth, $1.75, net. Flexible leather, $2.25, net. Lea Brothers & Co., Publishers, Philadelphia and New York.

Gonorrhea, stricture, chancroid and syphilis, with all of their complications and sequelæ, are discussed by the author in a thoroughly practical manner. In every instance the best approved methods of treatment are clearly and tersely outlined. The illustrations are good. The type, paper and binding are better than are usually found in a work of the size. R.

